# Cell cycle–dependent localization of the proteasome to chromatin

**DOI:** 10.1038/s41598-020-62697-2

**Published:** 2020-04-02

**Authors:** Yuki Kito, Masaki Matsumoto, Atsushi Hatano, Tomoyo Takami, Kiyotaka Oshikawa, Akinobu Matsumoto, Keiichi I. Nakayama

**Affiliations:** 1 Division of Cell Biology and Medical Institute of Bioregulation, Kyushu University, Fukuoka, Japan; 2 Division of Proteomics, Medical Institute of Bioregulation, Kyushu University, Fukuoka, Japan; 3 Department of Omics and Systems Biology, Niigata University Graduate School of Medical and Dental Sciences, Niigata, Japan; 4 YCI Laboratory for Trans-Omics, Young Chief Investigator Program, RIKEN Center for Integrative Medical Sciences, Kanagawa, Japan

**Keywords:** Proteasome, Proteomics

## Abstract

An integrative understanding of nuclear events including transcription in normal and cancer cells requires comprehensive and quantitative measurement of protein dynamics that underlie such events. However, the low abundance of most nuclear proteins hampers their detailed functional characterization. We have now comprehensively quantified the abundance of nuclear proteins with the use of proteomics approaches in both normal and transformed human diploid fibroblasts. We found that subunits of the 26S proteasome complex were markedly down-regulated in the nuclear fraction of the transformed cells compared with that of the wild-type cells. The intranuclear proteasome abundance appeared to be inversely related to the rate of cell cycle progression, with restraint of the cell cycle being associated with an increase in the amount of proteasome subunits in the nucleus, suggesting that the nuclear proteasome content is dependent on the cell cycle. Furthermore, chromatin enrichment for proteomics (ChEP) analysis revealed enrichment of the proteasome in the chromatin fraction of quiescent cells and its apparent dissociation from chromatin in transformed cells. Our results thus suggest that translocation of the nuclear proteasome to chromatin may play an important role in control of the cell cycle and oncogenesis through regulation of chromatin-associated transcription factors.

## Introduction

Most biological processes including development, cell differentiation and proliferation, and homeostasis in mammals are regulated at the level of gene expression. A full understanding of such processes will thus require comprehensive characterization of how the expression of each gene is regulated^1^. The combination of genomics, proteomics, and other molecular technologies with bioinformatics has recently led to a rapid increase in our knowledge of gene regulatory networks that control gene expression^2–4^. Proteomics is an indispensable technique for characterization of the dynamics of proteins. Given that the abundance of nuclear proteins that contribute to the control of gene expression is generally low, however, the application of proteomics to characterization of the dynamics of such proteins has been limited.

Various methods to enrich nuclear proteins have been developed in an attempt to overcome this limitation. Although subcellular fractionation is a typical approach that has long been applied to separate the cytoplasm and nucleus with the use of hypotonic buffers and detergents, it is not suitable for isolation of proteins that bind to chromatin. Chromatin enrichment for proteomics (ChEP) enriches interphase chromatin and allows quantitative analysis of chromatin binding proteins. This approach is based on the cross-linking of intranuclear proteins to DNA in hypotonic buffer followed by isolation of the protein-DNA complex by centrifugation as a transparent gelatinous pellet^5^. Another approach to nuclear protein enrichment based on the interaction of such proteins with synthetic DNA containing transcription factor response elements has been developed^6^. This approach is restricted to *in vitro* conditions and so evaluates only indirectly protein-DNA binding under physiological conditions. Although several other methods have been developed in recent years to interrogate chromatin binding proteins, a disadvantage of these methods is that non–chromatin-associated proteins cannot be completely eliminated.

The 26S proteasome complex featured in this study is a key component of the ubiquitin-proteasome system (UPS), which is responsible for the catabolism of many proteins in both the cytoplasm and nucleus. The UPS mediates two discrete steps in such catabolism: the covalent attachment of multiple ubiquitin molecules to the protein substrate by a ubiquitin-activating enzyme (E1), a ubiquitin-conjugating enzyme (E2), and a ubiquitin ligase (E3), and the degradation of the polyubiquitylated protein by the 26S proteasome complex^7,8^. In addition to the degradation of cytoplasmic proteins, the 26S proteasome regulates gene expression by controlling the abundance of transcription factors associated with chromatin^9–11^. The dynamics of proteasome localization have been well studied, with the 26S proteasome, which is formed by assembly of 20S and 19S complexes in the cytoplasm, being thought to translocate into the nucleus^12^. In yeast, the amount of the proteasome in the nucleus is greater in the stationary phase than in the growth phase^13,14^. On the other hand, the nuclear abundance of the proteasome in human cells is thought to increase in the proliferative phase, although many studies have been performed with cancer cells and the dynamics of the nuclear proteasome in normal human cells remain unknown^15^. In addition, analysis of the localization dynamics of the proteasome has often been performed with the use of proteasome subunits fused to a fluorescent protein, but whether such fusion influences incorporation of the subunit into the proteasome complex and its function has been unclear. Furthermore, evaluation of proteasome localization dynamics ideally requires a comprehensive analysis of all proteasome subunits, but such an analysis has been technically difficult to perform.

We have now developed a novel nuclear fractionation method to evaluate the network of nuclear proteins responsible for the control of gene expression. In this method, nuclei isolated by cell disruption with a hypotonic buffer are subjected to nucleolytic enzyme treatment and exposed to a solution of high ionic strength in order to allow the extraction and concentration of nuclear proteins without cytoplasmic contamination. The combination of this approach with label-free nontargeted proteomics showed that proteasome subunits disappeared from the nucleus of normal human cells in association with cell transformation. A detailed targeted proteomics analysis of proteasome subunits^16^ revealed the loss of all subunits in the nucleus of transformed cells. Further analyses suggested that the nuclear proteasome binds to chromatin in a cell cycle–dependent manner and may contribute to gene regulatory networks.

## Results

### Nuclear proteasome abundance declines in association with oncogenic transformation

**Figure 1:**
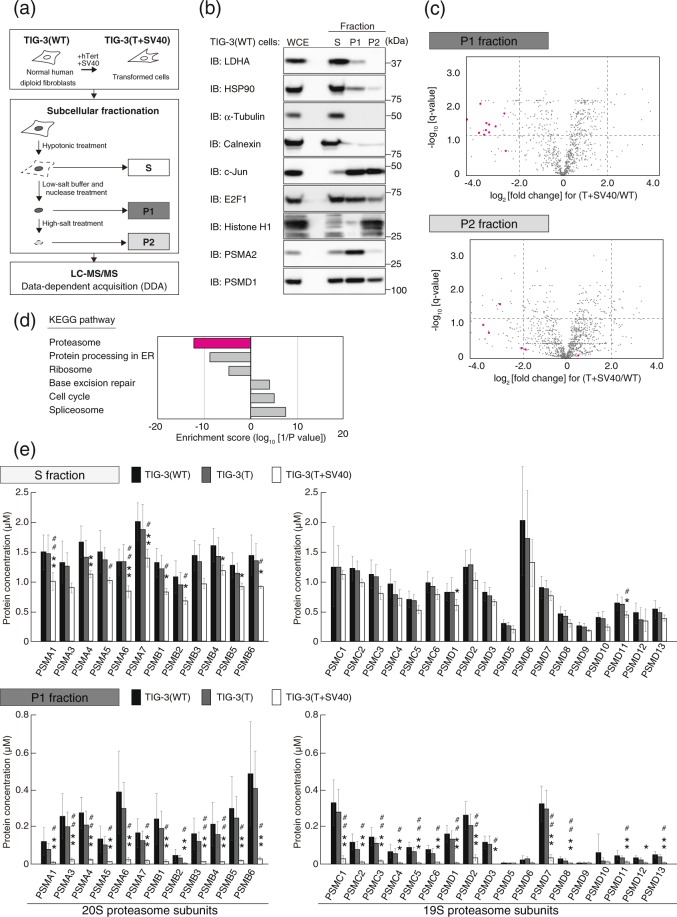
Proteomics analysis shows that the nuclear abundance of the proteasome is reduced in association with oncogenic transformation. (**a**) Strategy for cell transformation and subcellular fractionation. TIG-3(WT) cells were transformed by the introduction of hTert and the early region of SV40. Cellular proteins of both TIG-3(WT) cells and the transformed cells, designated TIG-3(T + SV40), were separated into three fractions corresponding to the cytoplasm (S), the nucleoplasm and proteins loosely associated with chromatin (P1), and proteins tightly associated with chromatin (P2). Each fraction was analyzed by liquid chromatography and tandem mass spectrometry (LC-MS/MS). (**b**) Validation of subcellular fractionation. Whole cell extract (WCE) and subcellular fractions of TIG-3(WT) cells were subjected to immunoblot (IB) analysis with antibodies to LDHA, HSP90, α-tubulin, and calnexin as cytoplasmic marker proteins; to c-Jun and E2F1 as nucleoplasmic marker proteins; to histone H1 as a chromatin marker protein; and to the proteasome subunits PSMA2 and PSMD1. (**c**) Label-free proteomics analysis of P1 and P2 fractions. The log_2_[fold change] for protein abundance in TIG-3(T + SV40) cells relative to TIG-3(WT) cells and the –log_10_[q-value] are shown as Volcano plots. The threshold for determining differential expression is indicated by the dashed lines (q-value of  ≤0.05). Proteasomal proteins are shown in red. (**d**) KEGG (Kyoto Encyclopedia of Genes and Genomes) pathway–based enrichment analysis of the P1 fraction with Fisher’s exact test. The top three significantly (*P*  <  0.05) up-regulated or down-regulated KEGG pathways in TIG-3(T + SV40) cells relative to TIG-3(WT) cells are shown. (**e**) Absolut**e** quantification of proteasome subunits by iMPAQT analysis. The S and P1 fractions of TIG-3(WT), TIG-3(T), and TIG-3(T + SV40) cells were analyzed for the abundance of proteasome subunits of both the 20S and 19S complexes. Data are means ± s.d. for six independent biological replicates. *P  <  0.05, **P < 0.01 compared with TIG-3(WT); ^#^*P* <  0.05, ^##^*P* <  0.01 compared with TIG-3(T) (one-way ANOVA followed by Bonferroni’s post hoc test).

We studied TIG-3 normal human diploid fibroblasts. These cells were engineered to stably express the human telomerase catalytic subunit (hTert) either alone or together with the simian virus 40 (SV40) early region, with the resulting cells being designated TIG-3(T) and TIG-3(T + SV40) and representing immortalized and transformed cells, respectively. To evaluate the dynamics of nuclear proteins that directly control gene expression, we developed a novel nuclear fractionation method and performed label-free quantitative proteomics analysis (Fig. 1a). Wild-type (WT) TIG-3 cells and TIG-3(T + SV40) cells were treated with a hypotonic buffer to allow separation of the nucleus (P fraction) from the cytoplasm (S fraction). The P fraction was treated with a nucleolytic enzyme in a low-salt solution and then centrifuged, and the resulting supernatant (P1 fraction) was collected whereas the pellet was incubated in a high-salt solution and then centrifuged to yield the P2 fraction. The validity of the fractionation was verified by immunoblot analysis of TIG-3(WT) cells (Fig. 1b). Proteins known to be located predominantly in the cytoplasm such as LDHA, HSP90, α-tubulin, and calnexin were enriched in the S fraction compared with the P1 and P2 fractions. Proteins that are present in the nucleoplasm or that are loosely associated with chromatin but liberated from chromatin by nuclease treatment, represented by the transcription factors c-Jun and E2F1, were detected in the P1 fraction. On the other hand, proteins that are strongly associated with chromatin such as histone H1 were recovered in the P2 fraction after exposure of the P1 fraction to the solution of high ionic strength. These results thus demonstrated the effectiveness of the fractionation method for the separation of nuclear proteins associated with chromatin.

The P1 and P2 fractions were then subjected to label-free proteomics analysis. Volcano plot analysis and KEGG pathway analysis^17^ of the quantitative proteomics data revealed that the abundance of multiple 26S proteasome subunits in the P1 fraction was reduced in TIG-3(T + SV40) cells compared with TIG-3(WT) cells (Fig. 1c,d), whereas that of most such subunits in the P2 fraction did not differ between the two cell types. The reduced abundance of proteins associated with the endoplasmic reticulum (ER) apparent in the P1 fraction of TIG-3(T + SV40) cells (Fig. 1d) might be attributable to differential contamination of the P1 fraction with cytoplasmic proteins, given that a residual amount of the ER protein calnexin was detected in the P1 fraction of TIG-3(WT) cells by immunoblot analysis (Fig. 1b). The 26S proteasome is composed of 33 subunits, and we prepared synthetic proteins corresponding to 30 of the subunits by stable isotope labeling with [^13^C_6_/^15^N_2_]Lys and [^13^C_6_/^15^N_4_]Arg and then performed absolute quantification of these subunits in the S and P1 fractions of TIG-3(WT), TIG-3(T), and TIG-3(T + SV40) cells by application of our recently developed large-scale targeted proteomics platform, designated iMPAQT (*in vitro* proteome–assisted MRM [multiple reaction monitoring] for protein absolute quantification)^16^. Precise and absolute quantification of 26S proteasome subunits by the iMPAQT system confirmed a marked decrease in the abundance of almost all analyzed subunits in the P1 fraction of TIG-3(T + SV40) cells compared with that of TIG-3(WT) cells (Fig. 1e). Such depletion of proteasome subunits was not apparent in the P1 fraction of TIG-3(T) cells.

**Figure 2:**
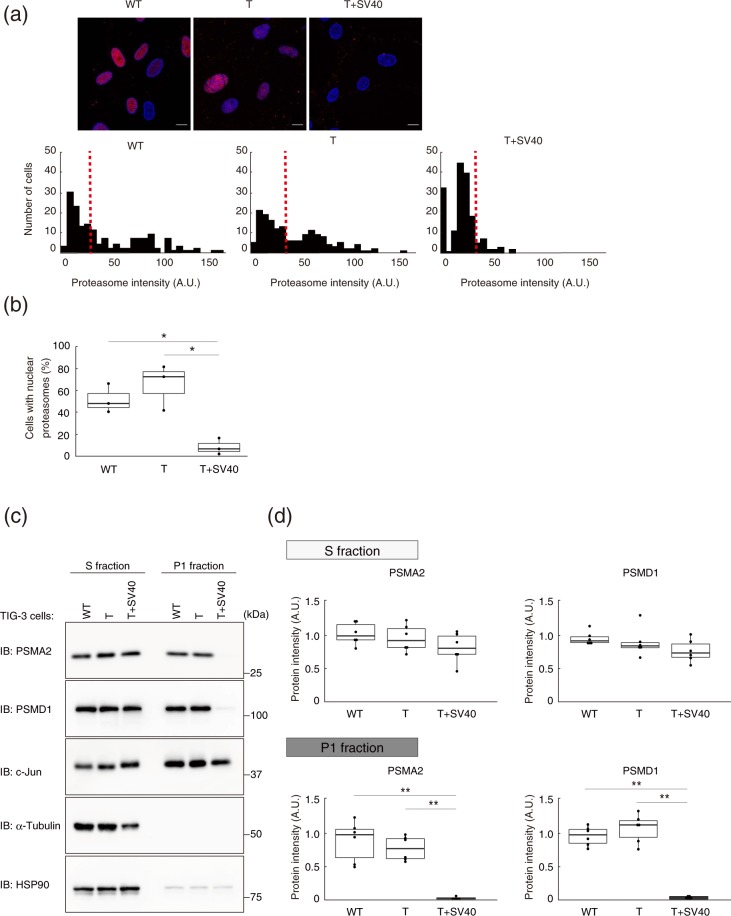
Immunofluorescence and immunoblot analyses confirming that the nuclear localization of the proteasome is suppressed by oncogenic transformation. (**a**) Immunofluorescence staining of α-proteasomal subunits (red) in TIG-3(WT), TIG-3(T), and TIG-3(T + SV40) cells fixed after cytoplasm removal. DNA (blue) was counterstained with DAPI (upper panel). Scale bars, 10 µm. Histograms show the frequency distribution for normalized immunofluorescence signal intensity (A.U., arbitrary units) in the nucleus, with the median value for TIG-3(WT) cells being indicated with a dashed red line (lower panel). (**b**) Box plot of the percentage of cells with proteasome fluorescence intensity greater than the median of the distribution for TIG-3(WT) cells determined as in (**a**). Data are for four independent biological replicates. *P < 0.05 (one-way ANOVA followed by Bonferroni’s post hoc test). (**c**) Immunoblot analysis of PSMA2 and PSMD1 in the S and P1 fractions of TIG-3(WT), TIG-3(T), and TIG-3(T + SV40) cells. HSP90 and α-tubulin were examined as markers for the S fraction, and c-Jun as a marker for the P1 fraction. (**d**) Quantitative analysis of band intensities for PSMA2 and PSMD1 in experiments similar to that in (**c**). Data are from six independent biological replicates. **P < 0.01 (one-way ANOVA followed by Bonferroni’s post hoc test). Boxes and whiskers show the median, the lower and upper quartiles, and the minimum and maximum values. R software version 3.6.2 (https://www.r-project.org) was used to draw the plots in (**b**) and (**d**).

We next examined the nuclear localization of the proteasome in the normal and transformed cells by immunofluorescence analysis with antibodies to α-proteasome subunits (Fig. 2a). The intensity of the nuclear immunofluorescence signal normalized by the area of 4′,6-diamidino-2-phenylindole (DAPI) staining of DNA was plotted as a frequency distribution, and cells with a value greater than the median of the distribution for TIG-3(WT) cells were defined as positive for nuclear localization of the proteasome. The ratio of nuclear proteasome–positive cells to total cells was significantly reduced for TIG-3(T + SV40) cells compared with TIG-3(WT) or TIG-3(T) cells (Fig. 2b). Immunoblot analysis confirmed that the amounts of two representative proteasome subunits, PSMA2 (α2 subunit of the 20S complex) and PSMD1 (non-ATPase regulatory subunit 1 of the 19S complex), were markedly reduced in the P1 fraction of TIG-3(T + SV40) cells compared with that of TIG-3(WT) or TIG-3(T) cells (Fig. 2c,d).

**Figure 3:**
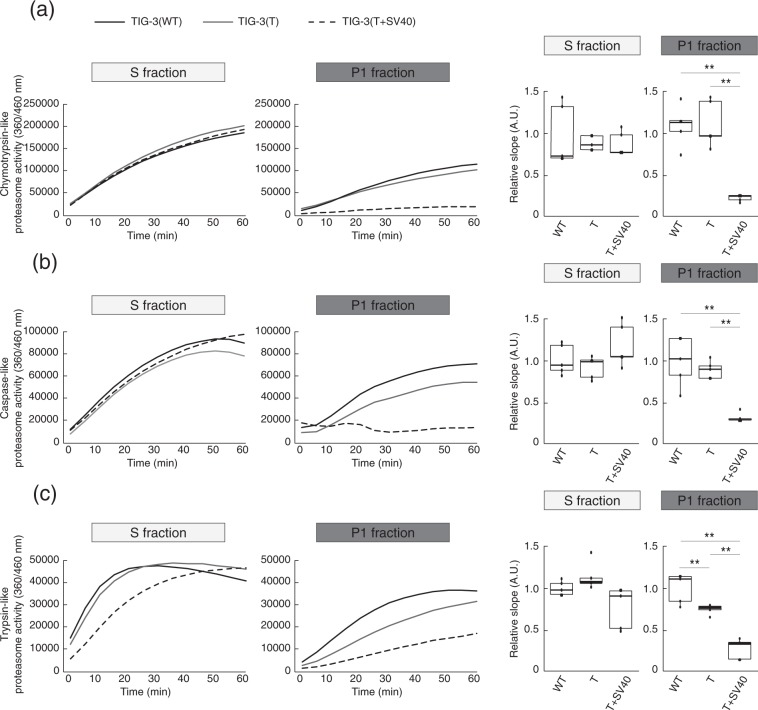
Nuclear proteasome activity changes with transformation in parallel with its abundance. Chymotrypsin-like (**a**), caspase-like (**b**), and trypsin-like (**c**) proteasome activities in S and P1 fractions of TIG-3(WT), TIG-3(T), and TIG-3(T + SV40) cells were determined on the basis of digestion of specific fluorogenic peptides. Representative reaction time courses and quantitation of activity based on the relative slope for 0 to 30 min for five independent biological replicates are shown. **P < 0.01 (one-way ANOVA followed by Bonferroni’s post hoc test). Boxes and whiskers show the median, the lower and upper quartiles, and the minimum and maximum values. R software version 3.6.2 (https://www.r-project.org) was used to draw the box plots.

We also measured proteasome activity in the S and P1 fractions of parental, immortalized, and transformed fibroblasts by determining the degradation rate for specific fluorogenic peptide substrates targeted by chymotrypsin-like (Fig. 3a), caspase-like (Fig. 3b), and trypsin-like (Fig. 3c) activities of the proteasome. The proteasome activity with each substrate in the P1 fraction was reduced for TIG-3(T + SV40) cells compared with TIG-3(WT) or TIG-3(T) cells, whereas the activity in the S fraction did not differ significantly among TIG-3(WT), TIG-3(T), and TIG-3(T + SV40) cells. These results suggested that the activity of the proteasome changed in parallel with its abundance. We confirmed that two proteasome inhibitors, MG-132 and epoxomicin^18,19^, blocked the chymotrypsin-like (Supplementary Fig. 1), caspase-like (Supplementary Fig. 2), and trypsin-like (Supplementary Fig. 3) activities in both P1 and S fractions of TIG-3(WT), TIG-3(T), and TIG-3(T + SV40) cells, suggesting that these activities were indeed attributable to the proteasome.

### Large T, but not small T, alters the nuclear abundance of the proteasome

**Figure 4:**
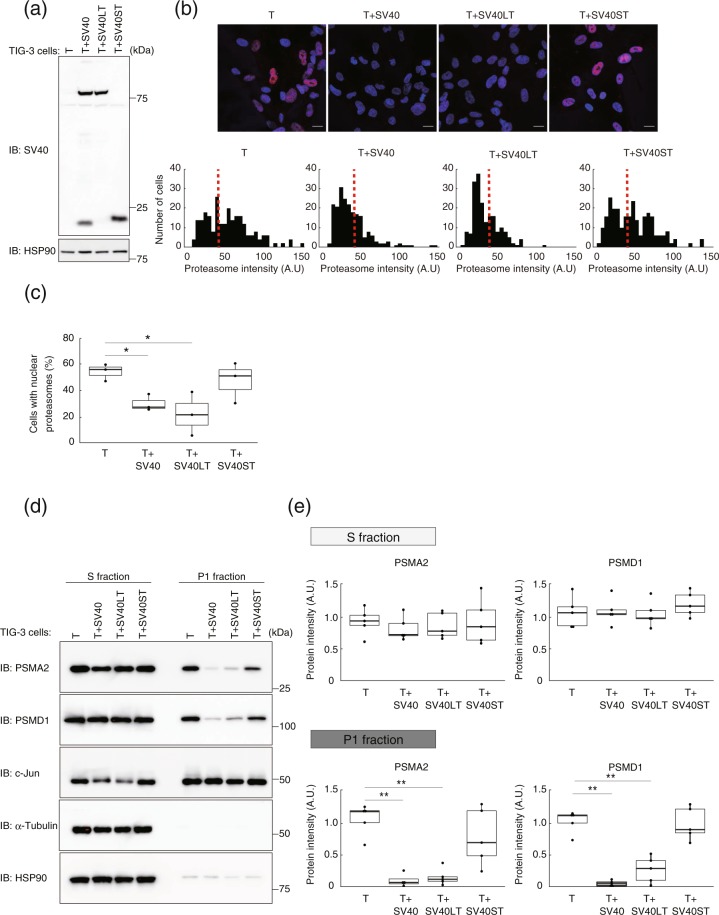
SV40 LT, but not ST, alters the nuclear abundance of the proteasome. (**a**) Immunoblot analysis of SV40 early region expression in whole cell extracts of TIG-3(T), TIG-3(T + SV40), TIG-3(T + SV40LT), and TIG-3(T + SV40ST) cells. The antibodies detect the NH_2_-terminal region of both LT and ST. (**b**) Immunofluorescence staining of α-proteasomal subunits (red) in cells as in (**a**). The cells were fixed after cytoplasm removal and before staining. DNA (blue) was counterstained with DAPI (upper panel). Scale bars, 20 µm. Histograms show the frequency distribution for normalized immunofluorescence signal intensity in the nucleus, with the median value for TIG-3(T) cells being indicated with the dashed red line (lower panel). (**c**) Box plot for the percentage of cells with a proteasome fluorescence intensity greater than the median of the distribution for TIG-3(T) cells determined as in (**b**). Data are for three independent biological replicates. *P < 0.05 (one-way ANOVA followed by Bonferroni’s post hoc test). (**d**) Immunoblot analysis of PSMA2 and PSMD1 in the S and P1 fractions of TIG-3(T), TIG-3(T + SV40), TIG-3(T + SV40LT), and TIG-3(T + SV40ST) cells. HSP90 and α-tubulin were examined as controls for the S fraction, and c-Jun as a control for the P1 fraction. (**e**) Quantitative analysis of band intensities for PSMA2 and PSMD1 in experiments similar to that in (**d**). Data are for five independent biological replicates. **P < 0.01 (one-way ANOVA followed by Bonferroni’s post hoc test). Boxes and whiskers show the median, the lower and upper quartiles, and the minimum and maximum values. R software version 3.6.2 (https://www.r-project.org) was used to draw the plots in (**c**) and (**e**).

The early region of the SV40 genome encodes both large T antigen (LT) and small T antigen (ST), which are generated by alternative splicing^20^. The introduction of this region of SV40 into cells has long been adopted as a model of cell transformation and early tumorigenesis^21,22^. LT binds to and inactivates the tumor suppressor proteins p53 and retinoblastoma protein (Rb), resulting in the promotion of cell cycle progression^23^. On the other hand, ST binds to protein phosphatase 2 A and inhibit its serine-threonine phosphatase activity^24^. To examine whether LT or ST affects the nuclear localization of the proteasome, we established TIG-3(T) cells expressing LT or ST, which were designated TIG-3(T + SV40LT) and TIG-3(T + SV40ST), respectively (Fig. 4a). Immunofluorescence analysis revealed that, compared with TIG-3(T) cells, the ratio of nuclear proteasome–positive cells to total cells was significantly reduced for TIG-3(T + SV40LT) cells to an extent similar to that apparent for TIG-3(T + SV40) cells, whereas no such a change was detected for TIG-3(T + SV40ST) cells (Fig. 4b,c). Similar results were obtained by immunoblot analysis of PSMA2 and PSMD1 in the P1 fraction (Fig. 4d,e). These findings thus indicated that the change in the nuclear localization of the proteasome apparent in TIG-3(T + SV40) cells is attributable to LT rather than to ST.

### Nuclear proteasome abundance changes with the cell cycle

**Figure 5:**
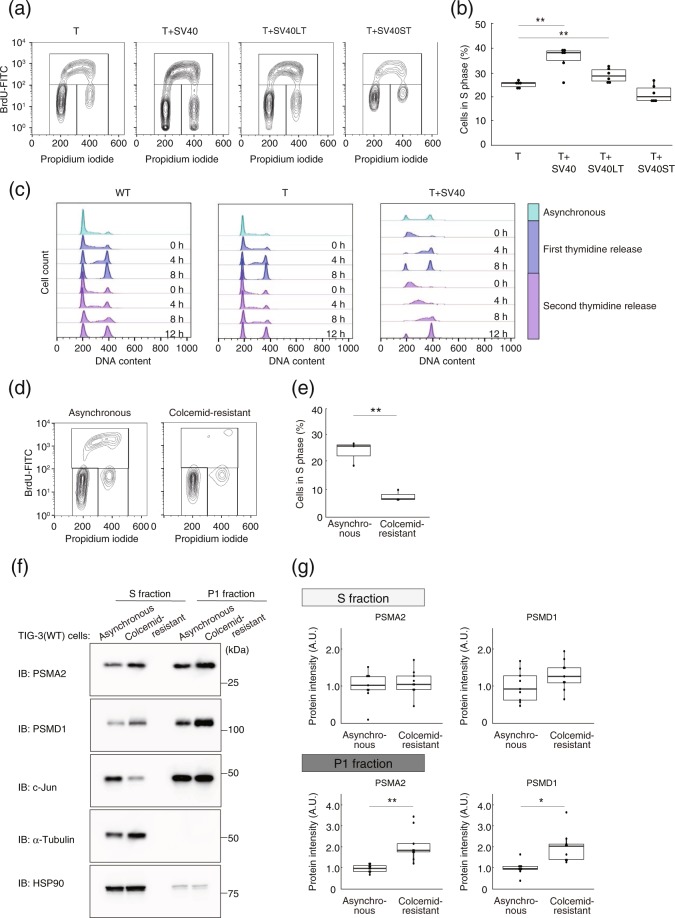
Nuclear proteasome abundance is increased in response to cell cycle arrest at G_0_-G_1_. (**a**) Cell cycle analysis in TIG-3(T), TIG-3(T + SV40), TIG-3(T + SV40LT), and TIG-3(T + SV40ST) cells. Cells were labeled with bromodeoxyuridine (BrdU) for 90 min, fixed, and stained with fluorescein isothiocyanate (FITC)–conjugated antibodies to BrdU. Nuclear DNA was stained with propidium iodide, and the cells were then analyzed by flow cytometry. (**b**) Box plot of the proportion of proliferative cells (cells in S phase) in experiments similar to that in (**a**). Data are for six independent biological replicates. **P < 0.01 (one-way ANOVA followed by Bonferroni’s post hoc test). (**c**) Analysis of cell cycle progression by double thymidine block. TIG-3(WT), TIG-3(T), and TIG-3(T + SV40) cells were arrested at the G_1_-S transition by exposure to excess thymidine and then released for the indicated times. They were then stained with propidium iodide for flow cytometric analysis of DNA content. Data from the asynchronous cells are also shown at the top. (**d**) Cell cycle analysis by the colcemid-challenge test. Asynchronous TIG-3(WT) cells and TIG-3(WT) cells remaining (colcemid-resistant cells) after long-term treatment with colcemid and shake-off of those arrested at G_2_-M phase were analyzed for cell cycle profile as in (**a**). (**e**) Box plot of the proportion of BrdU-positive cells in experiments similar to that in (**d**). Data are for three independent biological replicates. **P < 0.01 (Student’s *t* test). (**f**) Immunoblo*t* analysis of PSMA2 and PSMD1 in S and P1 fractions of asynchronous and colcemid-resistant TIG-3(WT) cells. HSP90 and α-tubulin were examined as controls for the S fraction, and c-Jun as a control for the P1 fraction. (**g**) The band intensities for PSMA2 and PSMD1 in experiments similar to that in (**f**) were quantified. Data are for nine independent biological replicates. *P < 0.05, **P < 0.01 (Student’s *t* test). Boxes and whiskers show the median, *t*he lower and upper quartiles, and the minimum and maximum values. R software version 3.6.2 (https://www.r-project.org) was used to draw the plots in (**b**), (**e**), and (**g**).

The cell cycle is accelerated in cells transformed with SV40, with LT expression being correlated with the rate of cell cycle progression^25^. We confirmed that the proportion of cells in S phase was increased for TIG-3(T + SV40) or TIG-3(T + SV40LT) cells compared with TIG-3(T) cells (Fig. 5a,b). To characterize cell cycle dynamics in detail, we applied a double thymidine block in TIG-3(WT), TIG-3(T), and TIG-3(T + SV40) cells (Fig. 5c). Whereas most TIG-3(T + SV40) cells progressed synchronously to M phase via S phase, TIG-3(WT) and TIG-3(T) cells included populations that either progressed to M phase via S phase (cycling cells) or which remained in G_1_ phase (noncycling cells). Given that TIG-3(WT) and TIG-3(T) cells also showed a biphasic frequency distribution for nuclear proteasome abundance, whereas TIG-3(T + SV40) cells did not include cells with a high nuclear proteasome content (Fig. 2a,b), we hypothesized that changes in cell cycle status might be related to the nuclear localization of the proteasome.

To examine whether nuclear proteasome abundance is increased in quiescent cells, we performed a colcemid-challenge test on TIG-3(WT) cells in order to remove cycling cells^26^. The cells were thus cultured in the presence of the mitotic inhibitor colcemid for 12 h on each of 5 days, after which those that had passed through mitosis were “shaken off.” The cells that had not passed through mitosis remained in the dish (colcemid-resistant cells), with this procedure thus resulting in enrichment of noncycling cells. We confirmed that the proportion of cells in S phase was greatly reduced among colcemid-resistant TIG-3(WT) cells compared with corresponding asynchronous cells (Fig. 5d,e). Immunoblot analysis revealed that the abundance of proteasome subunits in the P1 fraction was significantly greater for the colcemid-resistant cells than for the asynchronous cells (Fig. 5f,g). Collectively, our results thus suggested that the nuclear abundance of the proteasome is related to cell cycle status.

**Figure 6:**
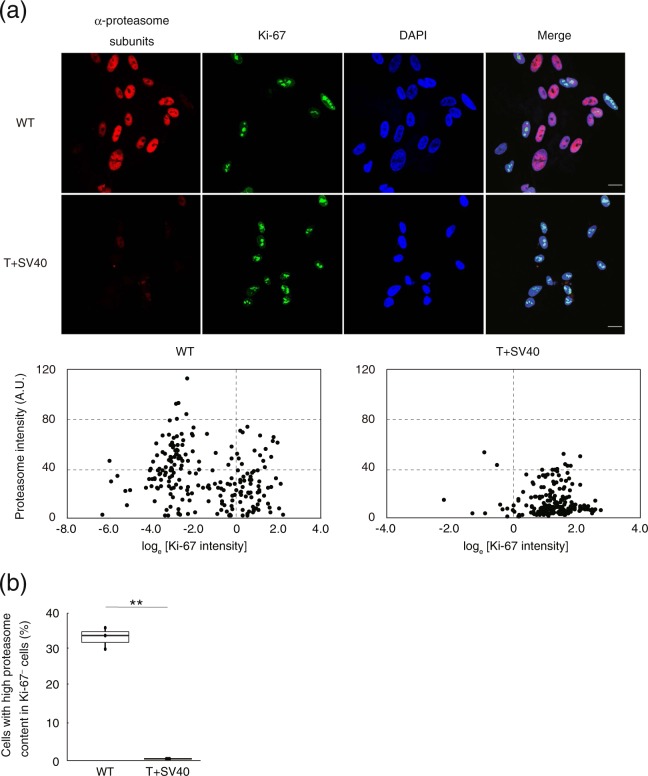
Immunofluorescence analysis of cell cycle–dependent proteasome localization. (**a**) Immunofluorescence staining of α-proteasomal subunits (red) and Ki-67 (green) in TIG-3(WT) and TIG-3(T + SV40) cells. DNA (blue) was counterstained with DAPI (upper panel). Scale bars, 20 µm. Scatter plots for the intensity of nuclear proteasome staining versus log_e_[Ki-67 staining intensity] are also shown (lower panel). (**b**) Box plot for the percentage of cells with high proteasome content in Ki-67^−^ cells (quiescent cells) determined as in (**a**). Data are for three independent biological replicates. **P < 0.01 (Student’s *t* test). Boxes and whiskers show the median, the lower and upper quartiles, and the minimum and maximum values. R software version 3.6.2 (https://www.r-project.org) was used to draw the plot in (**b**).

To directly demonstrate the increase in the nuclear abundance of the proteasome in quiescent cells, we performed immunofluorescence analysis for both the proteasome and the proliferation marker Ki-67 (Fig. 6a). For TIG-3(WT) cells, most Ki-67^+^ cells (proliferating cells) showed a low nuclear abundance of the proteasome, whereas Ki-67^–^ cells (quiescent cells) were positive for nuclear proteasome immunofluorescence. On the other hand, most TIG-3(T + SV40) cells were positive for Ki-67 (cycling) and showed few nuclear proteasome signals (Fig. 6b). These results thus supported the notion that the nuclear abundance of the proteasome is regulated in a cell cycle–dependent manner.

### Nuclear proteasomes localize to chromatin in a cell cycle–dependent manner

**Figure 7:**
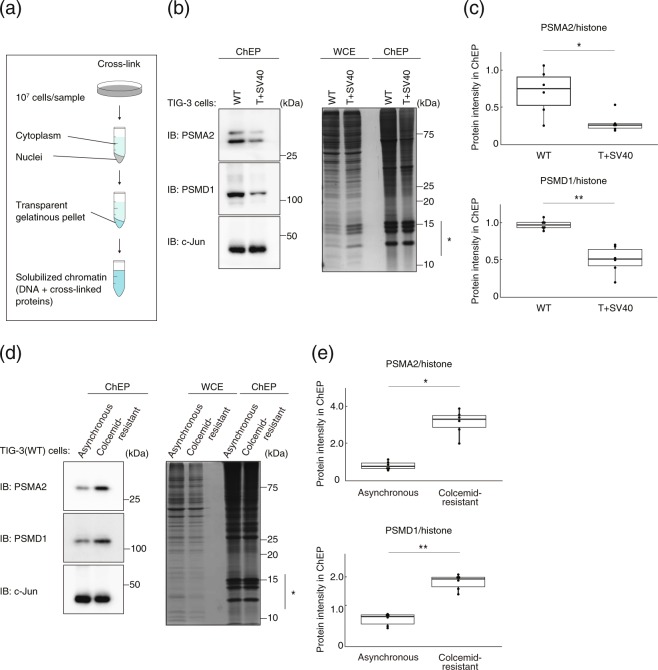
The nuclear proteasome is enriched in the chromatin fraction. (**a**) Scheme for isolation of the chromatin-binding protein fraction by ChEP. (**b**) Immunoblot analysis of PSMA2 and PSMD1 in the chromatin-enriched fraction of TIG-3(WT) or TIG-3(T + SV40) cells. c-Jun was examined as a positive control for the chromatin-enriched fraction (left panel). Total proteins in the whole cell extract (WCE) and the ChEP fraction were also revealed by Coomassie brilliant blue staining of the polyacrylamide gel (right panel). Note that histones (indicated by the asterisk) are enriched in the ChEP fraction. (**c**) The abundance of PSMA2 and PSMD1 in the ChEP fraction was quantified in experiments similar to that in (**b**). Data were normalized by histone abundance and are for six independent biological replicates. *P < 0.05, **P < 0.01 (Student’s *t* test). (**d**) Immunoblo*t* analysis of PSMA2 and PSMD1 in the chromatin-enriched fraction of asynchronous and colcemid-resistant (quiescent) TIG-3(WT) cells (left panel). Total proteins in the whole cell extract and in the ChEP fraction were also revealed by Coomassie brilliant blue staining of the polyacrylamide gel (right panel). (**e**) Quantitative analysis of PSMA2 and PSMD1 in the ChEP fraction for experiments similar to that in (**d**). Data are from six independent biological replicates. *P < 0.05, **P < 0.01 (Student’s *t* test). Boxes and whiskers show the median, *t*he lower and upper quartiles, and the minimum and maximum values. R software version 3.6.2 (https://www.r-project.org) was used to draw the plots in (**c**) and (**e**).

Nuclear proteasomes control gene expression by mediating the degradation of transcription factors associated with chromatin^9,10^. We therefore performed ChEP analysis of TIG-3(WT) and TIG-3(T + SV40) cells to determine whether the association of the proteasome with chromatin is dependent on the cell cycle (Fig. 7a). Such analysis revealed that the amount of the proteasome bound to chromatin was reduced in TIG-3(T + SV40) cells compared with TIG-3(WT) cells (Fig. 7b,c). Furthermore, the levels of PSMA2 and PSMD1 associated with chromatin were significantly higher in colcemid-resistant TIG-3(WT) cells than in corresponding asynchronous cells (Fig. 7d,e). These results thus suggested that nuclear proteasomes are bound to chromatin in quiescent cells but are liberated from chromatin on reentry into the cell cycle.

**Figure 8:**
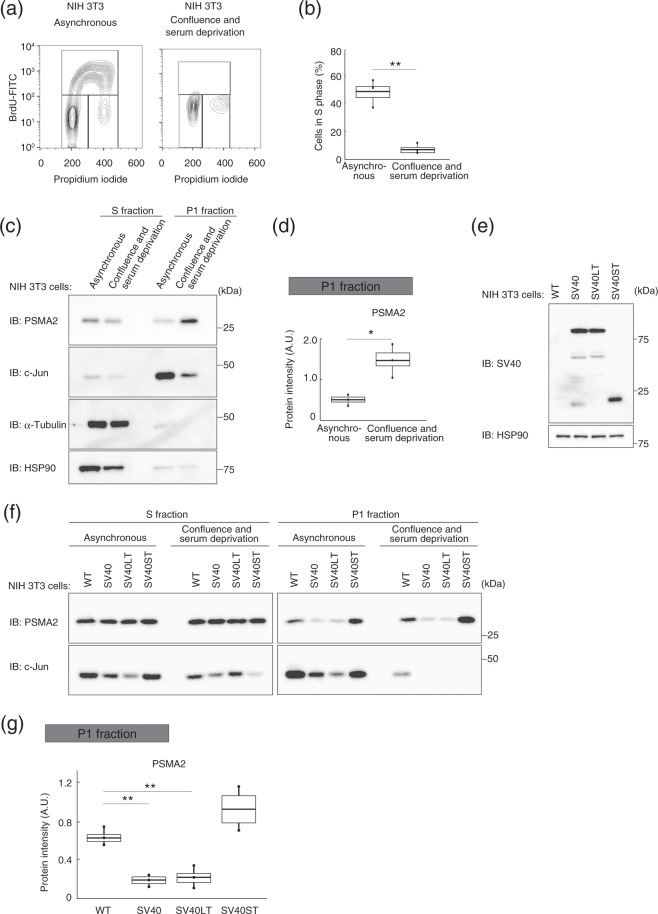
Nuclear proteasome abundance is regulated in a cell cycle–dependent manner in NIH 3T3 cells. (**a**) Cell cycle analysis of NIH 3T3 cells in asynchronous culture or after induction of quiescence by confluent culture and serum deprivation. Cells were labeled with BrdU for 30 min, fixed, and stained with FITC-conjugated antibodies to BrdU. Nuclear DNA was stained with propidium iodide, and the cells were then analyzed by flow cytometry. (**b**) Box plot of the proportion of proliferative cells (cells in S phase) for experiments similar to that in (**a**). Data are for three independent biological replicates. **P < 0.01 (Student’s *t* test). (**c**) Immunoblot analysis of PSMA2 in the S and P1 fractions of NIH 3T3 cells maintained as in (**a**). HSP90 **a**nd α-tubulin were examined as controls for the S fraction, and c-Jun as a control for the P1 fraction. (**d**) Quantitative analysis of PSMA2 in the P1 fraction for experiments similar to that in (**c**). Data are for three independent biological replicates. *P < 0.05 (Student’s *t* test). (**e**) Immunoblo*t* analysis of SV40 early region expression in NIH 3T3(WT), NIH 3T3(SV40), NIH 3T3(SV40LT), and NIH 3T3(SV40ST) cells. (**f**) Immunoblot analysis of PSMA2 in the S and P1 fractions of NIH 3T3(WT), NIH 3T3(SV40), NIH 3T3(SV40LT), and NIH 3T3(SV40ST) cells maintained as in (**a**). (**g**) Quantitative **a**nalysis of band intensities for PSMA2 in the P1 fraction of cells after induction of quiescence by confluent culture and serum deprivation for experiments similar to that in (**f**). Data are for three independent biological replicates. **P < 0.01 (one-way ANOVA followed by Bonferroni’s post hoc test). Boxes and whiskers show the median, the lower and upper quartiles, and the minimum and maximum values. R software version 3.6.2 (https://www.r-project.org) was used to draw the plots in (**b**), (**d**), and (**g**).

To confirm the generality of our findings with TIG-3 human fibroblasts, we performed similar experiments with NIH 3T3 mouse fibroblasts. The cell cycle of NIH 3T3 cells was arrested by confluent culture and serum deprivation for 96 h^27^. We confirmed that the proportion of cells in S phase was greatly reduced among such arrested NIH 3T3 cells compared with corresponding asynchronous cells (Fig. 8a,b). Immunoblot analysis revealed that the abundance of the proteasome in the P1 fraction was significantly greater for the arrested cells than for the asynchronous cells (Fig. 8c,d).

NIH 3T3 cells were also engineered to stably express the SV40 early region, LT, or ST, with the resulting cells being designated NIH 3T3(SV40), NIH 3T3(SV40LT), and NIH 3T3(SV40ST), respectively (Fig. 8e). Immunoblot analysis revealed that, compared with parental cells, the abundance of the proteasome in the P1 fraction was significantly reduced for NIH 3T3(SV40) and NIH 3T3(SV40LT) cells but not for NIH 3T3(SV40ST) cells (Fig. 8f,g). Together, these results thus suggested that the nuclear abundance of the proteasome is related to cell cycle status not only in human TIG-3 cells but also in mouse NIH 3T3 cells.

## Discussion

We have here developed a novel nuclear fractionation method that identifies nuclear proteins associated with chromatin by combining conventional subcellular fractionation with targeting of the chromatin binding proteome, and which allows a comprehensive characterization of nuclear proteins that contribute to gene regulatory networks. With this new method, we have shown that the nuclear abundance of the proteasome changes in a cell cycle–dependent manner.

The validity of a fractionation method is key to its application to analysis of the intracellular localization of molecules. We assessed the effectiveness of our method by immunoblot analysis of proteins in each of the obtained fractions as well as by immunofluorescence analysis. Given that the cytoplasmic proteins examined were largely absent from the P1 fraction, contamination appears to be less of a problem for our method than for conventional fractionation. In any case, to eliminate the effects of such potential cytoplasmic contamination of the P1 fraction, we performed targeted proteomics analysis.

Many proteins function as components of large complexes and thereby contribute to intracellular processes including regulation of gene expression^28^. In the present study, we applied our recently developed targeted proteomics approach, iMPAQT^16^, to the absolute quantification of almost all subunits of the 26S proteasome complex. This approach is able to simultaneously evaluate the abundance of all these subunits in a single sample. This technology should also prove to be applicable to other large protein complexes such as the ribosome or spliceosome, thereby allowing estimation of the stoichiometry of the subunits within each entire complex.

The UPS regulates transcriptional activity in various situations^9,29,30^, with the localization of the proteasome being a key determinant of such regulation^15,31^. Recent studies of proteasome localization have adopted live cell imaging techniques that allow tracing of the movement of proteasomal subunits tagged with fluorescent labels such as green fluorescent protein^13^. These techniques have revealed that, in *Saccharomyces cerevisiae*, proteasomes are included within proteasome storage granules (PSGs)^32,33^ that serve as storage compartments in a cell cycle–dependent manner. In the stationary phase, PSGs are initially localized on the nucleoplasmic side of the nuclear envelope as spherical organelles without a membrane, whereas they eventually translocate to the cytoplasm in cells that remain in stationary phase over the long term. On exit from long-term stationary phase, PSGs rapidly dissociate and the proteasomes are transported to the nucleus within minutes^31,32^. Similar phenomena in mammals have been detected only in melanoma cells and hippocampal neurons to date^13^. Although our fractionation-based proteomics approach did not distinguish between PSGs and chromatin-bound proteasomes in normal human fibroblasts, immunofluorescence analysis revealed that proteasomes appeared to be dispersed throughout the nucleus with no signs of PSG-like structures in these cells. However, it is difficult to formally exclude the possibility that detergent treatment in the immunostaining procedure precluded the detection of PSGs.

It has long been unclear whether degradation of transcriptional regulators by the UPS promotes or represses overall transcriptional activity^29,34,35^. High transcriptional activity is likely associated with an increase in proteasome activity, however, a phenomenon referred to as transcriptional license. For example, in the mammalian circadian clock, UPS-mediated degradation of the transcriptional repressors Cryptochrome (CRY) and Period (PER) activates transcription of the genes for the clock proteins CLOCK and BMAL1^36,37^. Importantly, the proteasome degrades different substrates in quiescent and proliferating cells. In the present study, we found that, in normal human fibroblasts, chromatin-bound proteasomes accumulate in the noncycling state but decrease in abundance in the cycling state. The identification of specific substrates of the nuclear proteasome in the quiescent phase awaits future study.

Many proteins that contribute to cell cycle progression such as cyclins as well as cyclin-dependent kinases and their inhibitors, as well as the tumor suppressor protein p53, signaling-related proteins such as IκBα, transcriptional factors, and misfolded and malfunctioning proteins, are targets of the UPS^30^. For maintenance of quiescence, proteasomes actively degrade many substrates including proteins related to the Notch signaling pathway and the transcriptional regulator STAT5^38,39^. We previously showed that c-Myc undergoes degradation by the UPS in a manner dependent on the ubiquitin ligase SCF^Fbxw7^ in order to maintain stem cells in the quiescent state^40–42^. SCF^Fbxw7^ also regulates apoptosis by targeting MCL1 for ubiquitylation and destruction^43^. Proteasomes are recruited to chromatin and degrade the transcriptional repressor ATF3 during the response to DNA damage^40^. This degradation of ATF3 results in the recruitment of RNA polymerase II to gene promoters and activation of transcription^11^. Ubiquitylation of RAG-2 mediated by SCF^Skp2^ at the G_1_-to-S transition links degradation of the V(D)J recombinase to the cell cycle^44^. The nuclear proteasome thus maintains protein homeostasis in the nucleus by degrading specific substrates in response to changes in environmental and cellular conditions^45,46^. However, it is likely that many such specific substrates remain to be identified.

SV40 LT binds to and inactivates the tumor suppressor proteins p53 and Rb, resulting in the promotion of cell cycle progression^23^, and in induction of restriction point failure^47^. In mammals, temporary cell cycle arrest and entry into quiescence occur before cells reach the environmental factor–dependent restriction point of G_1_ phase^48–50^. We now show that cells transformed with SV40 and therefore unable to maintain quiescence as a result of the inactivation of p53 and Rb function manifested a reduction in the abundance of chromatin-bound proteasomes. This effect is likely a result of active cell cycling in the transformed cells rather than a direct consequence of loss of a specific function of p53 or Rb. On the other hand, the nuclear abundance of the proteasome was previously found to be increased in tumor cells compared with normal cells^51,52^. Our experimental model in the present study is based on the expression of hTert and SV40 LT, which inactivates p53 and Rb function. The transformed cells may thus reflect the initial phase of carcinogenesis^53–55^, whereas the previous studies were based on clinical cancer specimens or cells established from cancer that might be expected to reflect a more advanced state of malignancy. It will thus be of interest to determine the nuclear abundance of the proteasome in more highly malignant cells expressing c-Myc or mutant Ras in addition to hTert and SV40 LT.

The mechanism by which nuclear proteasome abundance changes in association with cell cycle status or cell transformation remains to be determined. From the opposite perspective, it remains unclear precisely how dissociation of the proteasome from chromatin might contribute to cell cycle progression or carcinogenesis. The binding of the proteasome to chromatin would be expected to regulate gene expression through the degradation of transcription factors, but identification of the cellular processes regulated in such a manner awaits further study. If release of the proteasome from chromatin during cell cycle progression is essential for cancer growth and metastasis, characterization of the underlying mechanism may provide a basis for the development of new anticancer drugs.

## Methods

### Cell culture, transfection, and infection

Cell culture, transfection, and infection were performed as previously described^16,56,57^. TIG-3 human embryonic lung diploid fibroblasts (Japanese Collection of Research Bioresources) were cultured under an atmosphere of 5% CO_2_ at 37 °C in Dulbecco’s modified Eagle’s medium (DMEM) supplemented with 10% fetal bovine serum (Life Technologies) and antibiotics^57^. NIH 3T3 cells were cultured under the same conditions in DMEM supplemented with 10% fetal calf serum (Life Technologies) and antibiotics^58^. We authenticated both TIG-3 and NIH 3T3 cells and tested them for mycoplasma contamination. For retroviral infection, the mouse ecotropic retrovirus receptor was introduced into TIG-3 cells with the use of an amphotropic virus produced by HEK293T cells transfected with both pCX4-EcoVR and pGP/pE-ampho (Takara Bio)^16^. TIG-3 cells expressing the mouse ecotropic retrovirus receptor were infected with a retrovirus encoding hTert^56^. The resulting TIG-3(T) cells were infected with a retrovirus for the SV40 early region, SV40 LT, or SV40 ST and subjected to selection to yield TIG-3(T + SV40), TIG-3(T + SV40LT), or TIG-3(T + SV40ST) cells, respectively. Corresponding NIH 3T3 cells were established in the same way.

### Cell fractionation and sample preparation

Cells (1 × 10^7^) were suspended in 1.0 ml of a hypotonic solution containing 10 mM HEPES-NaOH (pH 7.6), 10 mM KCl, 1.5 mM MgCl_2_, 10% glycerol, 0.34 M sucrose, 1 mM phenylmethylsulfonyl fluoride, 3 μM aprotinin, and 20 μM leupeptin (all chemicals were from Sigma-Aldrich unless stated otherwise), and they were maintained on ice for 30 min before the addition of Nonidet P-40 (NP-40) and RNase A (Wako) to final concentrations of 0.5% and 0.1%, respectively, and incubation at room temperature for 15 min. The cell suspension was then centrifuged at 800 × *g* for 5 min at 4 °C, and the resulting supernatant was collected as the S fraction. The pellet was suspended in hypotonic buffer containing 0.5% NP-40, and the suspension was centrifuged again at 800 × *g* for 5 min. The resulting pellet was suspended in 0.2 ml of a low-salt buffer containing 25 mM Tris-HCl (pH 7.4), 2 mM MgCl_2_, 0.1% NP-40, and 0.5% Benzonase nuclease (Millipore) and incubated at 37 °C for 30 min before centrifugation at 2300 × *g* for 5 min at 4 °C. The resulting supernatant was collected as the P1 fraction, and the pellet was suspended in 0.2 ml of a solution containing 2 M KCl, 25 mM Tris-HCl (pH 7.4), and 0.5% Triton X-100 and maintained on ice for 30 min before centrifugation at 20,000 × *g* for 5 min at 4 °C. The resulting supernatant was collected as the P2 fraction.

For label-free quantification and MRM analysis, SDS was added to a final concentration of 1% to each fraction and each mixture was incubated at 95 °C for 10 min. The protein concentration of the samples was determined with the BCA assay (Bio-Rad). Cysteine residues were blocked by incubation of the samples with 2 mM tris(2-carboxyethyl)phosphine hydrochloride (Thermo Fisher Scientific) for 30 min at 37 °C followed by alkylation with 10 mM 2-iodoacetamide for 30 min at room temperature. The samples were then digested with trypsin (5 μg/ml, Thermo Fisher Scientific) for 14 h at 37 °C.

### Label-free quantification of proteins by LC-MS/MS analysis

Peptides were acidified by the addition of trifluoroacetic acid to a final concentration of 1% and were then subjected to LC-MS/MS analysis in DDA mode with a QExactive instrument coupled with a nano-LC system (AdvanceLC, Michrom). Up to the top 10 most abundant ions with a charge of 2+ or 3+ were selected from the survey scan with an isolation window of 1.6 *m/z* units and were fragmented by higher-energy collision dissociation with a normalized collision energy of 27. Acquired data were analyzed with MaxQuant (version 1.2.0.11) and the human IPI database (version 3.68; 87,083 entries)^59^.

### MRM analysis

Recombinant proteasome subunits were generated by *in vitro* transcription and translation with the use of a wheat germ extract and corresponding human cDNA clones. The purified proteins were digested with trypsin and subjected to LC-MS/MS analysis, and MS/MS spectra assigned to peptides with more than six amino acids were selected from the iMPAQT-knowledge database and converted into MRM transitions^16^. High-responding proteotypic peptide sequences were concatenated into several synthetic artificial protein sequences, and corresponding cDNAs were synthesized (Eurofins Genomics). Artificial protein sequences and corresponding MRM assays are listed in Supplementary Table 1. To obtain isotopically labeled recombinant proteins with maximum labeling efficiency, we generated expression strains by P1 transduction of *Escherichia coli* BL21(DE3) that was auxotrophic for arginine and lysine and was derived from *argA* and *lysA* deletion mutants in the Keio Collection of single-gene knockouts^60^. Recombinant proteins were expressed as His_6_-tagged molecules in the auxotrophic cells cultured in the presence of [^13^C_6_/^15^N_2_]Lys and [^13^C_6_/^15^N_4_]Arg and were purified with Ni-resin. The vector sequences for production of the recombinant proteins are listed in Supplementary Table 2. Both pENTR2B containing each vector sequence and pET21b-QcodeAA-DEST are recombined by the LR reaction in the bacterial cells. MRM analysis was performed with a QTRAP5500 instrument (SCIEX) operated in positive-ion mode as described previously^16^.

### Immunoblot analysis

Immunoblot analysis was performed as described previously^61–63^. Primary antibodies included those to PSMA2 (Cell Signaling Technology, 2455), to PSMD1 (Abcam, 140682), to LDHA (Cell Signaling Technology, 3582), to c-Jun (Cell Signaling Technology, 9165), to histone H1 (Merck Millipore, 05–457), to E2F1 (Cell Signaling Technology, 3742), to SV40 LT and ST (Santa Cruz Biotechnology, sc-148), to HSP90 (BD Transduction Laboratories, 610419), and to α-tubulin (Thermo Fisher Scientific, 13–8000), and to calnexin (Medical and Biological Laboratories, M178–3). Horseradish peroxidase–conjugated goat antibodies to mouse or rabbit immunoglobulin G were obtained from Promega^62^, and ECL reagents were from Thermo Fisher Scientific. Immune complexes were quantified with a ChemiDoc Imaging System (Bio-Rad). Uncropped blot images are provided in Supplementary Figures 4 to 7.

### Immunofluorescence analysis

Immunofluorescence analysis was performed as previously described^64–67^, with a minor modification. After cytoplasm was removed from cells by exposure to phosphate-buffered saline (PBS) containing 0.5% Triton X-100 and 4% polyethylene glycol, nuclei were fixed for 30 min at room temperature with 4% paraformaldehyde in PBS, incubated first with antibodies to α subunits of the 20S proteasome (Enzo Life Sciences, HP810) and to Ki-67 (Lab Vision Corporation, SP6) and then with Alexa Fluor 546– or Alexa Fluor 488–conjugated secondary antibodies (Thermo Fisher Scientific) in PBS containing 0.5% Triton X-100, 2% bovine serum albumin, 0.2% Tween 20, 10% glycerol, and 0.05% NaN_3_, and then covered with a drop of Vectashield Mounting Medium with DAPI (Vector Laboratories) before examination with a confocal fluorescence microscope (LSM700, Carl Zeiss)^64^. Proteasome fluorescence intensity relative to the nuclear area was quantified with the use of ImageJ software (NIH).

### Measurement of proteasomal activity

The *in vitro* assay of 26S proteasome activities was performed as previously described^68^. ATP and dithiothreitol were added to S and P1 fractions at final concentrations of 2 and 1 mM, respectively, and the resulting samples (200 μl, containing ~15 µg of total protein) were transferred to the wells of a 96-well microtiter plate (Optiplate-96, PerkinElmer) and mixed with 10 µl of fluorogenic substrate. The substrates included Z-Leu-Leu-Glu-AMC (final concentration of 75 μM, Enzo) for measurement of caspase-like activity, Suc-Leu-Leu-Val-Tyr-AMC (final concentration of 75 μM, Enzo) for measurement of chymotrypsin-like activity, and Boc-Leu-Arg-Arg-AMC (final concentration of 2.5 μM, Enzo) for measurement of trypsin-like activity. Fluorescence (excitation at 380 nm, emission at 460 nm) was monitored every 5 min for 1 h at 37 °C with a microplate fluorometer (EnSpire, PerkinElmer). Proteasome inhibitors were added to S and P1 fractions 10 min before the assay. MG-132 was obtained from Peptide Institute, and epoxomicin was from Selleck Chemicals. MG-132 was used at a concentration of 25 µM. Epoxomicin was used at a concentration of 1 nM for inhibition of chymotrypsin-like activity and at 100 nM for inhibition of caspase-like and trypsin-like activities. The slope of the initial linear portion of each reaction (0 to 30 min after reaction onset) was determined for comparison of proteasome activities.

### Experimental model for cell cycle arrest

An experimental model for cell cycle arrest was performed as described previously^69,70^. Cells were synchronized at G_1_-S with a double thymidine block. They were thus incubated in medium containing 2 mM thymidine for 18 h, released for 8 h, and exposed again to 2 mM thymidine for 16 h. The cells were collected at 0 h (G_1_-S phase), 4 h (S phase), 8 h (S-G_2_ phase), or 12 h (G_2_-M phase) after the first or second release.

### Colcemid-challenge test

A colcemid-challenge test were performed as previously described^26^. In brief, TIG-3 cells were treated with 2 mM thymidine for 18 h, released for 8 h, and incubated with 40 nM colcemid (*N*-desacetyl-*N*-methylocolchicine, Thermo Fisher Scientific) for 12 h, after which mitotic cells were removed by shake-off treatment. To ensure complete removal of mitotic cells, we repeated the shake-off process five times (40 nM colcemid for 12 h, release of cells for 12 h). The remaining cells were studied as colcemid-resistant cells.

### Serum deprivation and density-dependent growth inhibition

NIH 3T3 cells were rendered quiescent as previously described^27^. The cells were thus cultured in the presence of 0.5% calf serum for 96 h after achieving confluence.

### ChEP analysis

The protocol for cross-linking and purification of protein-DNA complexes was described previously^5,71^. In brief, cells (1 × 10^7^) were fixed by incubation for 10 min at 37 °C with 5 ml of 1% formaldehyde in PBS, after which glycine was added to a final concentration of 0.25 M and the cells were incubated for an additional 10 min at room temperature. The cells were harvested and resuspended in 1 ml of an ice-cold lysis buffer containing 25 mM Tris-HCl (pH 7.4), 0.1% Triton X-100, 85 mM KCl, and Roche protease inhibitors (one tablet per 10 ml of buffer solution)^5^, and the resulting lysates were centrifuged to obtain a nuclear pellet. The pellet was washed with 250 μl of lysis buffer supplemented with 0.4% RNase A (Wako). For removal of noncovalently bound proteins, the nuclei were suspended in 250 μl of a solution containing 50 mM Tris-HCl (pH 7.4), 10 mM EDTA, and 4% SDS before the addition of 3 volumes of a solution containing 10 mM Tris-HCl (pH 7.4), 1 mM EDTA, and 8 M urea. The nuclei were then isolated by centrifugation, suspended in 250 µl of a solution containing 10 mM Tris-HCl (pH 7.4), 1 mM EDTA, 25 mM NaCl, and 10% glycerol, and subjected to ultrasonic treatment, and the supernatant obtained after subsequent centrifugation was collected as the ChEP sample containing sheared, cross-linked chromatin.

### Cell cycle analysis by flow cytometry

Sample preparation and analysis were performed as described previously^72–74^. For determination of BrdU incorporation, cells were exposed to BrdU (10 μM) for 90 min, separated by centrifugation, suspended in PBS, and fixed with 70% ethanol at −20 °C^73^. They were again separated by centrifugation, incubated with FITC-conjugated mouse antibodies to BrdU (BD Transduction Laboratories, 556028) for 1 h at room temperature, and washed, after which DNA was stained with propidium iodide (10 μg/ml) diluted in PBS containing 0.1% Triton X-100 and RNase A (0.2 mg/ml). The samples were analyzed with a FACSCalibur flow cytometer and FlowJo v10 software (Becton Dickinson). The percentage of cells in S phase was calculated by fitting the propidium iodide signal to a cell cycle distribution with the Watson pragmatic model approach.

### Statistical analysis

Data were compared with Student’s *t* test or by one-way analysis of variance (ANOVA), with *P* values being adjusted by the Bonferroni method for multiple comparisons. All statistical analysis was performed with R software. A *P* value of <0.05 was considered statistically significant.

## Supplementary information

Supplementary information.

Supplementary information2.

Supplementary information3.

## Data Availability

The MS data have been deposited with the ProteomeXchange Consortium (http://proteomecentral.proteomexchange.org) via the JPOST partner repository under the data set identifiers PXD016259 (label-free quantification) and PXD016260 (MRM analysis).
