# Supplementary material for: Cell cycle–dependent localization of the proteasome to chromatin
Source: Sci Rep. 2020 Apr 2;10:5801. doi: 10.1038/s41598-020-62697-2 (PMC7118148; doi:10.1038/s41598-020-62697-2)
Supplement: Supplementary file 1 — Supplementary information. [file 41598_2020_62697_MOESM1_ESM.pdf]

Supplementary Information

Cell cycle–dependent localization of the proteasome to chromatin

Yuki Kito, Masaki Matsumoto, Atsushi Hatano, Tomoyo Takami, Kiyotaka Oshikawa, Akinobu Matsumoto & Keiichi I. Nakayama

Supplementary Figure Legends

Supplementary Figures 1–7

Supplementary Figure Legends

Supplementary Figure 1 | Proteasome inhibitors block digestion of the fluorogenic peptide substrate Suc-Leu-Leu-Val-Tyr-AMC. Chymotrypsin-like activity in the S and P1 fractions of TIG-3(WT), TIG-3(T), and TIG-3(T+SV40) cells was measured in the presence of dimethyl sulfoxide (DMSO) vehicle, 25 µM MG-132, or 1 nM epoxomicin. Representative reaction time courses (**a**) and reaction rates determined on the basis of the slope from 0 to 30 min for five independent biological replicates (**b**) are shown.The average of the slopes for each fraction of TIG-3(WT) cells treated with DMSO was defined as 1. $^{*}P < 0.05$, $^{**}P < 0.01$ (one-way ANOVA followed by Bonferroni's post hoc test). R software version 3.6.1 (https://www.r-project.org) was used to draw the plots in (**b**).

Supplementary Figure 2 | Proteasome inhibitors block digestion of the fluorogenic peptide substrate Z-Leu-Leu-Glu-AMC. Caspase-like activity in the S and P1 fractions of TIG-3(WT), TIG-3(T), and TIG-3(T+SV40) cells was measured in the presence of DMSO vehicle, 25 µM MG-132, or 100 nM epoxomicin. Representative reaction time courses (**a**) and reaction rates determined on the basis of the slope from 0 to 30 min for five independent biological replicates (**b**) are shown.The average of the slopes for each fraction of TIG-3(WT) cells treated with DMSO was defined as 1. $^{*}P < 0.05$, $^{**}P < 0.01$ (one-way ANOVA followed by Bonferroni's post hoc test). R software version 3.6.1 (https://www.r-project.org) was used to draw the plots in (**b**).

Supplementary Figure 3 | Proteasome inhibitors block digestion of the fluorogenic peptide substrate Boc-Leu-Arg-Arg-AMC. Trypsin-like activity in the S and P1 fractions of TIG-3(WT), TIG-3(T), and TIG-3(T+SV40) cells was measured in the presence of DMSO vehicle, 25 µM MG-132, or 100 nM epoxomicin. Representative reaction time courses (**a**) and reaction rates determined on the basis of the slope from 0 to 30 min for five independent biological replicates (**b**) are shown.The average of the slopes for each fraction of TIG-3(WT) cells treated with DMSO was defined as 1. $^{*}P < 0.05$, $^{**}P < 0.01$ (one-way ANOVA followed by Bonferroni's post hoc test). R software version 3.6.1 (https://www.r-project.org) was used to draw the plots in (**b**).

Supplementary Figure 4 | Full scans of key immunoblots in Figure 1b (**a**) and Figure

2c (**b**).

Supplementary Figure 5 | Full scans of key immunoblots in Figure 4a (**a**), Figure 4d (**b**), and Figure 5f (**c**).

Supplementary Figure 6 | Full scans of key immunoblots in Figure 7b (**a**) and Figure 7d (**b**) as well as of Coomassie blue–stained gels in Figure 7b (**c**) and Figure 7d (**d**).

Supplementary Figure 7 | Full scans of key immunoblots in Figure 8c (**a**), Figure 8e (**b**), and Figure 8f (**c**).

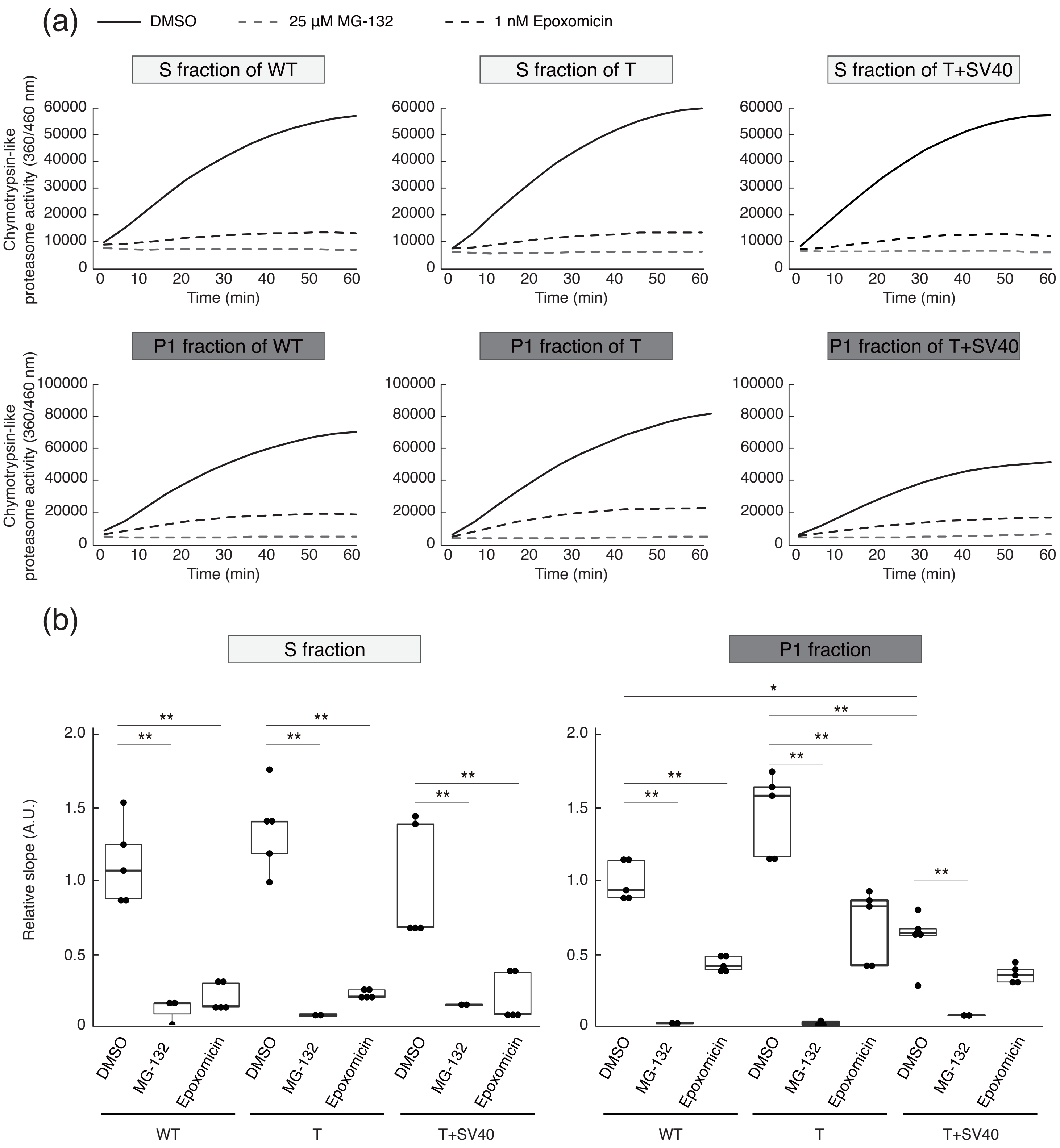

Kito et al. Supplementary Figure 1

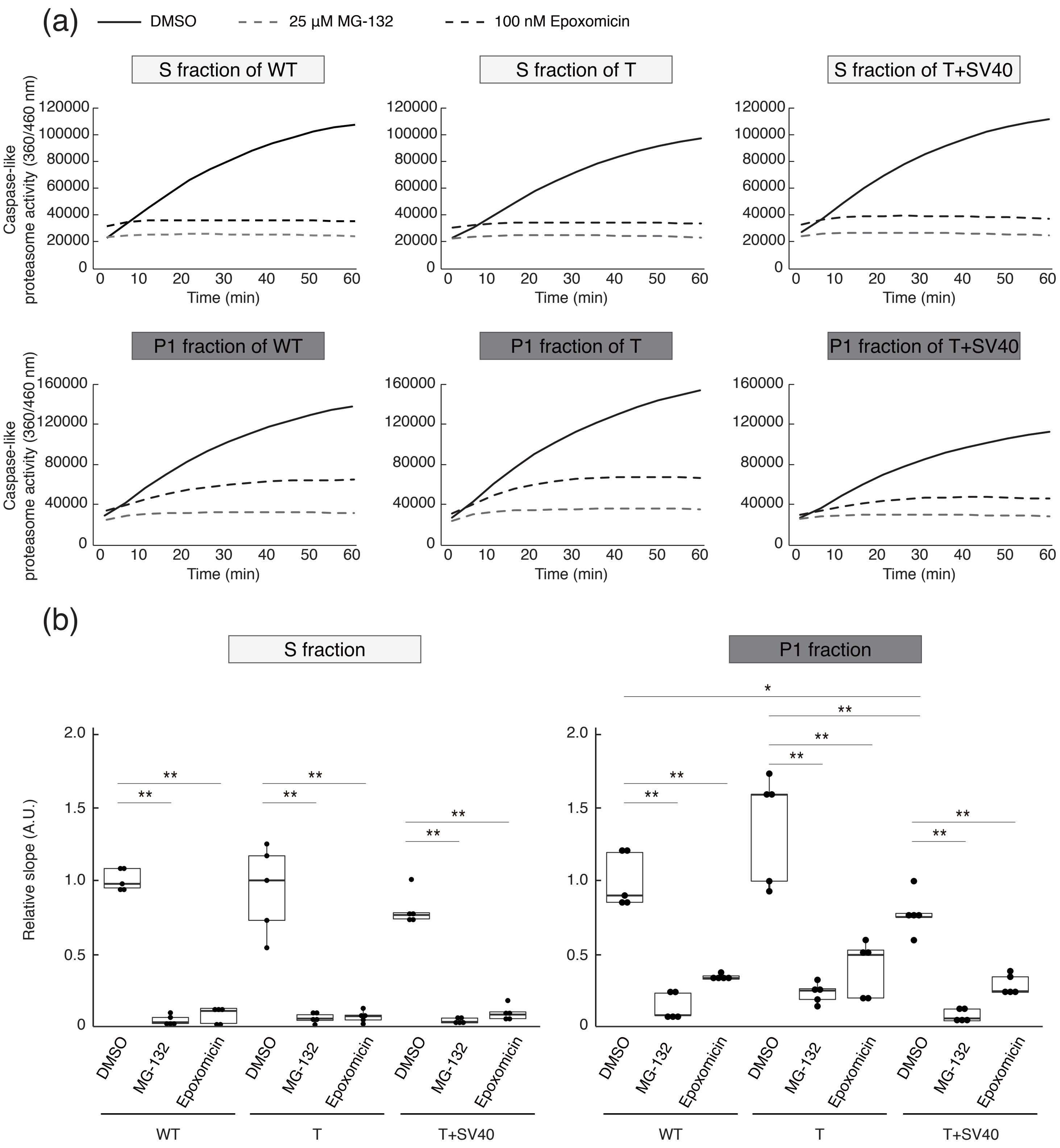

Kito et al. Supplementary Figure 2

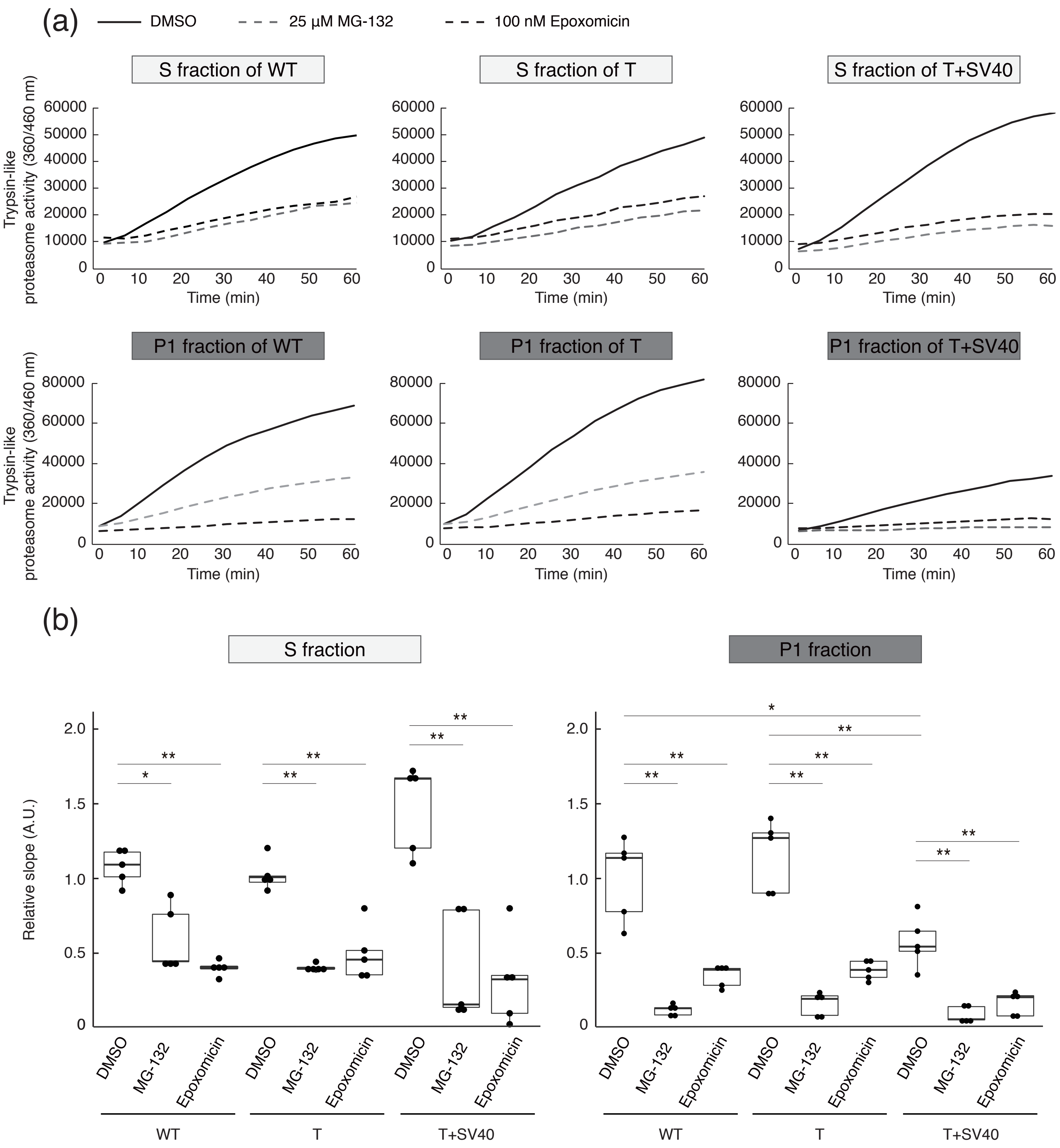

Kito et al. Supplementary Figure 3

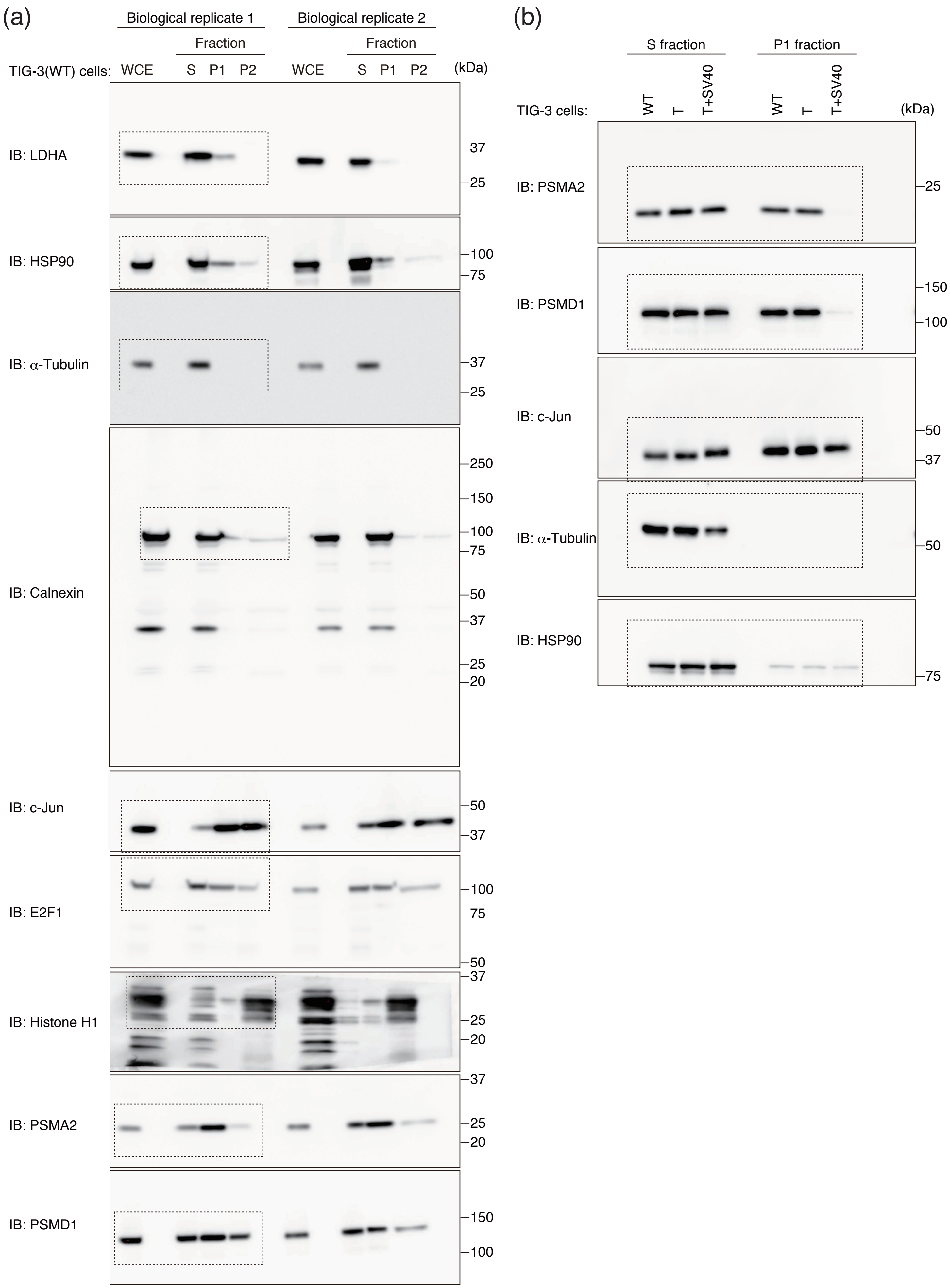

Kito et al. Supplementary Figure 4

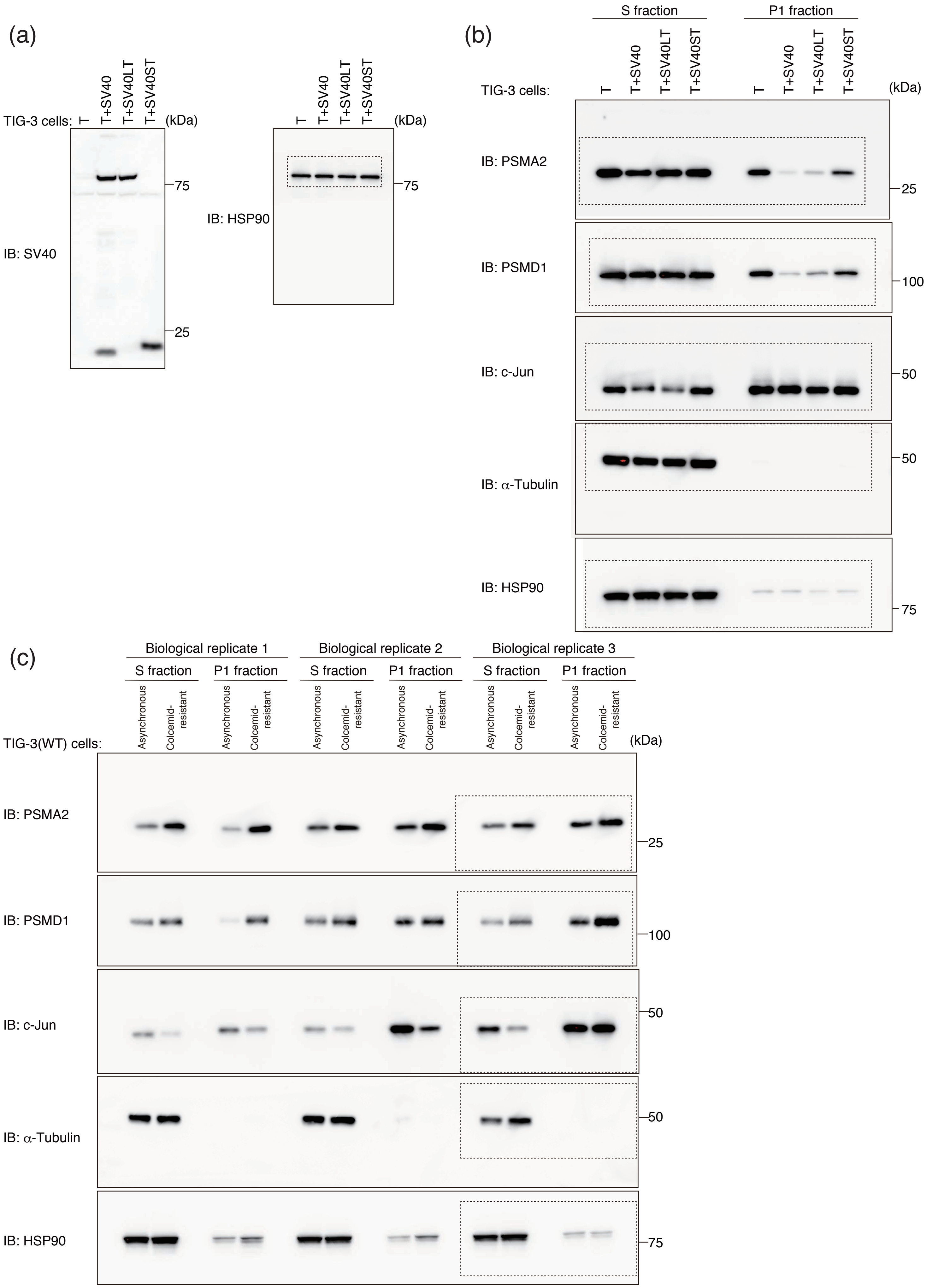

Kito et al. Supplementary Figure 5

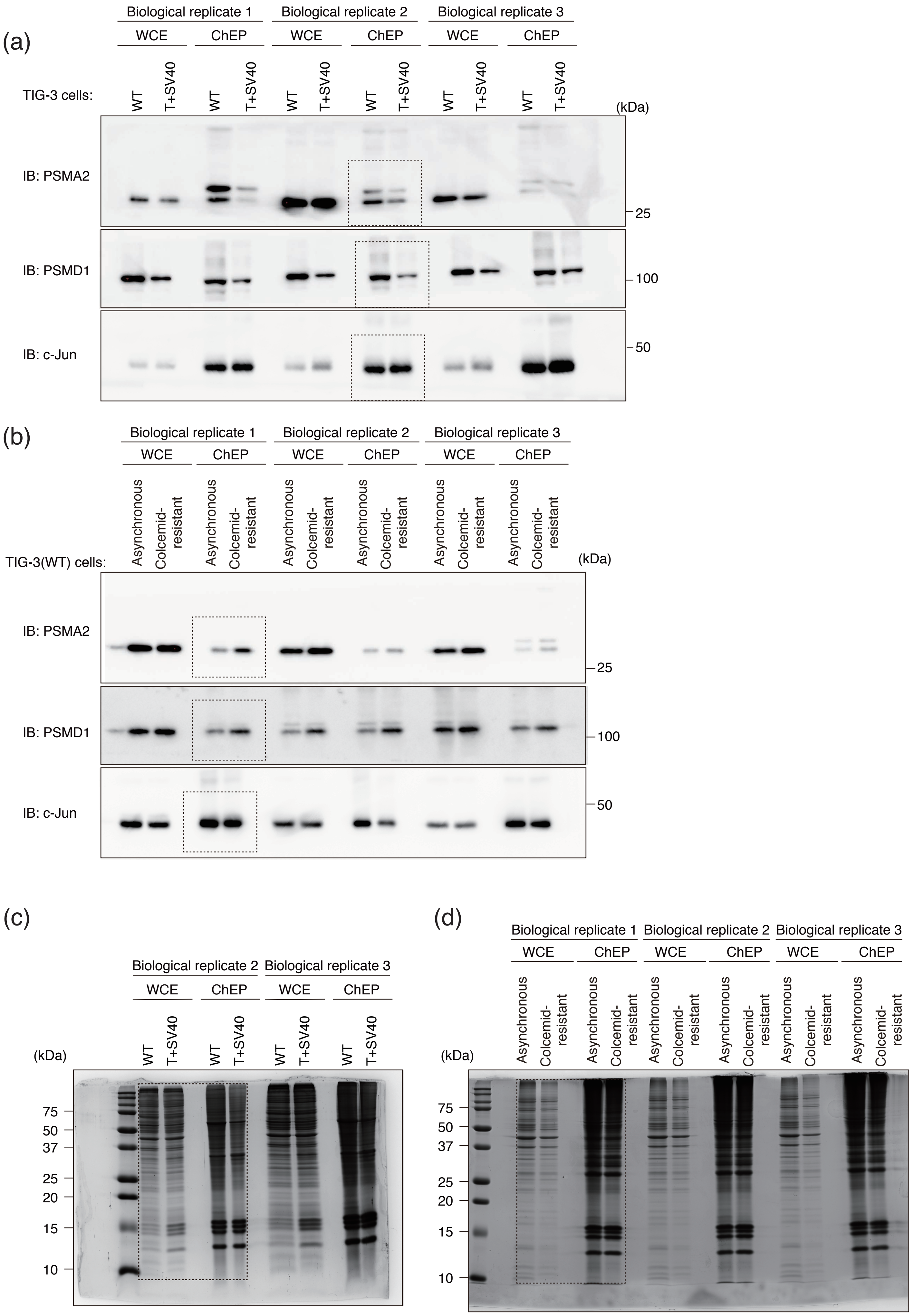

Kito et al. Supplementary Figure 6

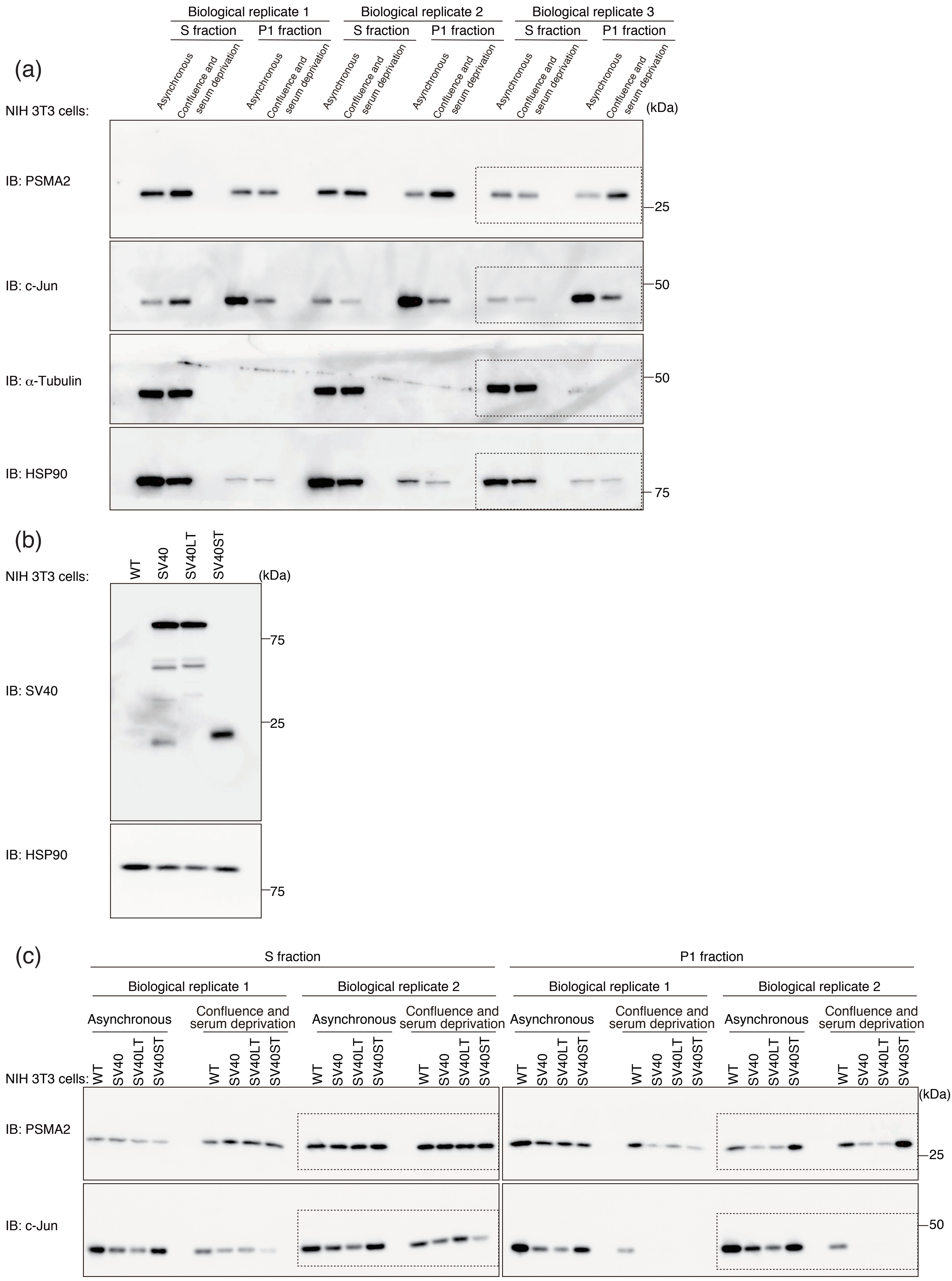

Kito et al. Supplementary Figure 7
